# Tensile Strength of Artificially Cemented Sandstone Generated via Microbially Induced Carbonate Precipitation

**DOI:** 10.3390/ma14164735

**Published:** 2021-08-22

**Authors:** Charalampos Konstantinou, Giovanna Biscontin, Fotios Logothetis

**Affiliations:** Department of Engineering, University of Cambridge, Cambridge CB2 1PZ, UK; gb479@cam.ac.uk (G.B.); fotlogo@gmail.com (F.L.)

**Keywords:** bio-cementation, sandstone, indirect tensile strength, fracture mechanics, fracture surface, grain size

## Abstract

Artificially bio-cemented sands treated with microbially induced calcite precipitation are weakly cemented rocks representing intermediate materials between locked and carbonate sands. Variations in cementation significantly affect the strength of sample, particularly tensile stregth. The modes of fracture and the surface characteristics resulting from the indirect tensile strength tests (Brazilian tests) are strongly correlated with the specimen strength and consequently the degree of cementation. This study examines the tensile strength of bio-cemented fine and coarse sands (average particle diameter 0.18 and 1.82 mm, respectively) and investigates failure modes by recording fracture evolution at both sides of specimen and surface characteristics of the reconstructed surfaces. The dimensionless slope parameter $Z_{2}$ provided the best fit with respect to tensile strength while the power spectral density was a good indicator of surface anisotropy. Finally, wavelet decomposition allowed for comparison of fracture surface characteristics of the two sands ignoring the grain size effects.

## 1. Introduction

Reproduction of rock specimens has received great attention in the literature [1,2,3,4], due to difficulties often experienced with conventional sampling. Synthetic material preparation allows structural parameters to be varied independently, and for their effects to be isolated [5].

One of the methods used to create rock-like material and more specifically bio-cemented sands, is microbially induced carbonate precipitation (MICP) [6]. This is a bio-cementation technique where calcium carbonate precipitates and acts as a cementation agent across sand particles. Bacteria hydrolise urea according to the first chemical equation and, in the presence of a calcium source, calcium carbonate precipitates into solid form (second chemical equation) [7,8].

$$\begin{array}{c} CO{\left(NH_{2}\right)}_{2}+2H_{2}O⟶2N{H_{4}}^{+}+C{O_{3}}^{2-} \end{array}$$

$$\begin{array}{c} Ca^{2+}+C{O_{3}}^{2-}⟶CaCO_{3} \end{array}$$

The method is used due to its relative ability to retain soil permeability [9] while enhancing the strength of the generated material. These artificially cemented sands are very weak rocks and represent a transitional material between soils and rocks, having characteristics in common with both [10,11]. Therefore, their behaviour ranges between that of sand and competent sandstone [12], depending mainly on the degree of cementation.

Studies of the synthetic sandstones created with MICP [9,13,14,15,16] show that the degree of cementation, largely controlled by the protocol followed for preparation, has the most influence on the resulting mechanical properties. Unconfined compressive strength (UCS) has been the most studied, with values reaching up to 12 MPa [9,17,18,19,20,21,22,23,24]. Other studies have focused on Poisson’s ratio or shear stiffness through measurements of shear wave velocity [24,25]. The tangent Young’s moduli, friction angle and cohesion were evaluated for several degrees of cementation through triaxial testing in a number of studies [9,26,27].

Research on the assessment of the tensile strength of the specimens prepared with MICP is very limited, mostly focusing on reinforcement characteristics of bio-cemented sands [28,29]. Brazilian splitting tests on specimens with varying relative densities were carried out by van Paassen [15]. The tensile strength ranged from 110 kPa to 620 kPa, while the ratio of Brazilian test strength (BTS) over UCS was less than 10%. An exponential curve provided the best fit for their results with respect to relative density.

The indirect tensile strength (or Brazilian) test is a widely used method to derive the tensile strength of rock samples because of its simple protocol. Apart from tensile stress–strain curves, the roughness of the failure surface itself also provides insight into the strength of tested samples, type of rock tested, specimen heterogeneity, and the nature of the failure mechanisms [30]. Surface roughness measurements can be correlated to the joint roughness coefficient (JRC), which is used to evaluate the shear behaviour of rock joints [3,31]. JRC is typically evaluated by visual comparison of the surface under examination with Barton’s standards JRC profiles [32] and is one of the very first tools for assessing strength through roughness.

Several methods can be used to characterise a surface. In metrology, the most common approach is to assess parameters describing classical morphology, by deriving characteristics based on the height-elevation variations [33,34,35,36]. Myers [37] proposed a dimensionless measure of roughness using the root mean square of the first derivative of a surface profile, $Z_{2}$, initially for gauging the light-scattering properties of a surface. Tse and Cruden [38] found that, among all the topological parameters, the dimensionless parameter $Z_{2}$ and the structure function (SF) showed the best correlation. These parameters were originally developed for 1D profiles, but have been extended to surfaces (2D) in recent years [39,40].

Surface roughness can also be evaluated in the frequency domain with the use of power spectral density, defined as the inverse Fourier transform of the autocorrelation function. The power spectral density provides an indication of the contributions of different frequencies to the overall roughness. This type of analysis has been extensively implemented in other fields, but not often in rock mechanics [41,42,43,44,45,46].

Finally, wavelet analysis has been used for characterising the nature of fluid flow over rough surfaces [47,48]. As in Fourier analysis, this analysis decomposes a signal into a summation of basis functions. The main difference between wavelet and Fourier analysis is that the former uses wavelets (derived from translations and dilations of a fixed function called the mother wavelet) while the latter uses trigonometric polynomials [49]. The advantage of the wavelet method is that roughness can be evaluated at different scales.

The main objectives of this study are: (i) the assessment of tensile strength of two MICP-treated sands, differing greatly in grain size, at various strengths or cementation levels, (ii) the quantification of the characteristics of the generated fracture surfaces during the indirect splitting tensile strength test and (iii) the correlation of the tensile strength of these specimens with the characteristics of the generated fracture surfaces.

Surface topology analysis can be a powerful tool to provide an indication of material strength, as well as information on the mode and conditions of failure. Several methods for calculating surface roughness available in the literature are applied to assess the most accurate and appropriate approach for this application.

## 2. Experimental Work and Methodology

### 2.1. Preparation of MICP-Treated Specimens

**Figure 1:**
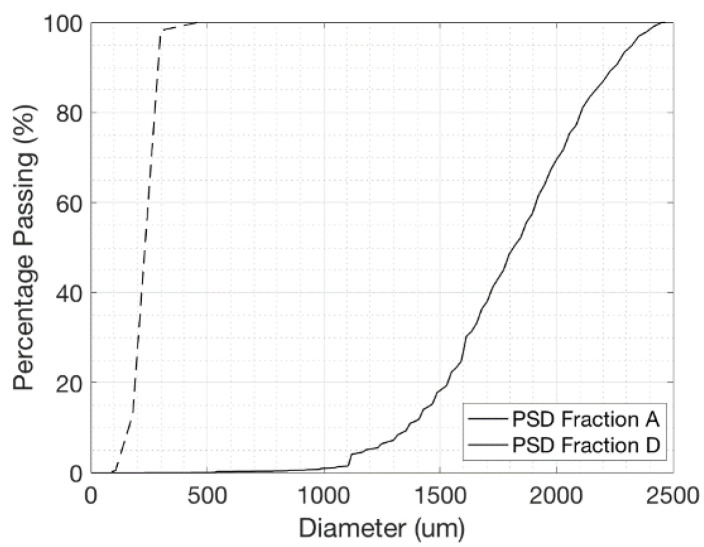
Particle size distribution curves for fine and coarse sands.

Fine and coarse sands, with mean particle diameters of 0.18 mm–1.82 mm, respectively, were treated with MICP to produce various cementation levels. The particle size distribution (PSD) of the base sands is shown in Figure 1. The Leighton Buzzard, UK, sand was provided washed, dried and free from organics, clay or silt. Silica (${\mathrm{SiO}}_{2}$) constituted more than 98% of the sand composition. The sand content of iron and aluminium oxides was low. The diameter of the final specimens was 70 mm with a height of 150 mm.

A detailed description of the MICP protocol followed to produce the specimens is provided in Konstantinou et al. [13,50], Konstantinou and Biscontin [51], Konstantinou [52], Wang et al. [53]. A two-stage process was used, consisting of an injection with bacterial solution in phase I and multiple injections of cementation solution in phase II. In each injection, 330 mL of solution was introduced to the specimen. The solutions were allowed to percolate into the specimens via gravity from the top and retained for 24 h in all cases. The bacteria solution was only injected once, at the beginning of the process. The previous solution was removed before each new injection.

The bacterial species selected was *Sporosarcina pasteurii* [54,55]. The growing medium consisted of 20 g/L yeast extract, 20 g/L agar, 10 g/L ammonium sulphate, and 0.13 M Tris buffer (base). After 24 h of incubation at 30 °C, the culture was stored at 4 °C. The bacterial colonies were introduced into liquid nutrient medium without agar, which was then placed in a shaking incubator for 24 h, forming the bacterial solution for injection. The optical density of the bacterial population at a wavelength of 600 nm, $OD_{600}$, was 1.5–2.0, measured on bacterial suspension samples of 3 mL using a visible light spectrophotometer.

The cementation solution utilised in this work consisted of 0.375 M urea, 0.25 M calcium chloride (CaCl_2_), and 3 g/L nutrient broth.

Different levels of cementation were obtained by varying the number of injections of cementation solution. The required injections number to achieve a targeted cementation level was defined as the total volume of solution required to be introduced in order to achieve a specific amount of carbonate precipitation overthe volume of one injection (330 mL).

Once the process was complete, the samples were carefully extracted from the moulds with minimal disturbance and then trimmed. The samples were sliced to form cylindrical specimens of smaller thickness (between 20 and 40 mm).

### 2.2. Calcium Carbonate Measurements

The calcium carbonate content was measured according to the ASTM standard method [56]. In a chamber with an attached pressure gauge, 30 mL of hydrochloric acid (HCl) 2.5 mol was introduced to 30 g of dried and ground sample. The carbonate (CaCO_3_) was dissolved and carbon dioxide was released according to the reaction:

$$CaCO_{3(s)}+2HCl_{\left(aq\right)}⟶CaCl_{2(aq)}+CO_{2(g)}+H_{2}O_{\left(l\right)}$$

The pressure reading was correlated to the amount of carbon dioxide and therefore the amount of carbonate that was dissolved. The degree of cementation was defined as the percentage of the weight of carbonate over the total weight of the sample tested.

### 2.3. Brazilian Tests

Indirect splitting tensile strength tests, or Brazilian tests, were conducted following ASTM [57] to assess the tensile strength. The cylindrical specimens had a 70 mm diameter and a thickness between 20 and 40 mm, or 0.2–0.8 times the diameter (as the standard specifies). A summary of the conducted tests is shown in Table 1.

Two cameras were used to capture the fracture onset and evolution on each side of the specimens. The front camera was capturing at 60 frames per second, while the second camera was recording at 30 frames per second.

### 2.4. Reconstruction of the Surfaces

**Figure 2:**
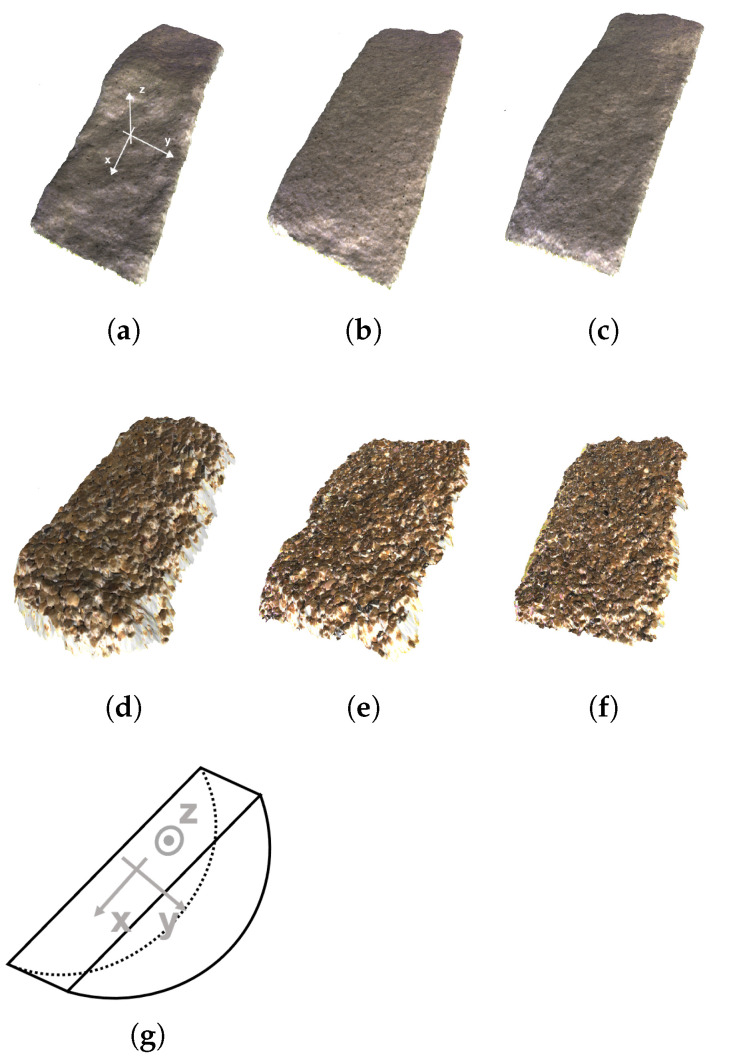
(**a**–**c**) Example reconstructions of fine sand surfaces: from weakest to strongest specimen (cementation levels at 3.1, 8.3 and 10.9%, respectively) (**b**–**f**) Example reconstructions of coarse sand surfaces (cementation levels at 3.9, 8.5 and 10.8%, respectively). The coordinate system is defined in (**g**) and in reconstruction (**a**): the *x*-axis runs along the diameter of the sample, the *y*-axis runs along the thickness of the specimen, and the *z*-axis represents the height variations of the surface.

A detailed 3D reconstruction of the surfaces was carried out using the Photometric Stereo method of Mecca et al. [58], Logothetis et al. [59]. This involves taking multiple images with different illumination using a specific LED configuration and then inferring the surface shape through a variational optimization on the inverse shading problem. The resulting reconstruction is a detailed triangle mesh with millions of vertices and precision of around 50 μm. Figure 2 shows three surfaces of fine and three surfaces of coarse sands in a digitised form.

**Figure 3:**
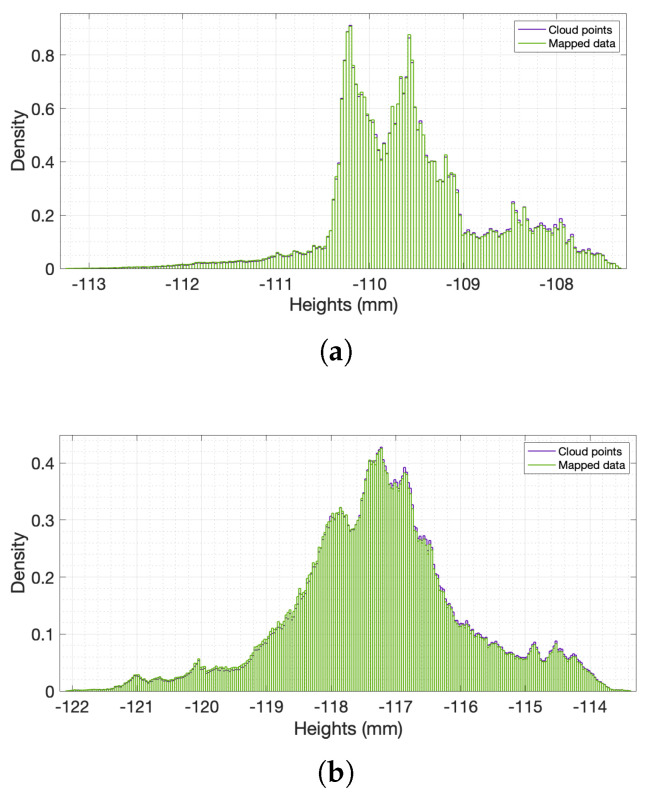
Examples of histograms of (**a**) fine sands at 7.3% cementation and (**b**) coarse sands at 6% cementation: the cloud points’ and the mapped data’s probability density functions (pdf’s) were plotted and normal pdf’s were fitted to compare the statistical characteristics. Density is defined as counts of a height measurement divided by the total number of data points.

The cloud points were then mapped onto a grid with 50 μm spacing using gridfit, a MATLAB^®^ function [60] which produces a surface describing the input data points as closely as possible. The algorithm splits each cell into triangles and interpolates linearly within each triangle to assign an elevation at the nodes. The smoothness of the final interpolating function can be defined by the user. In this case, a low smoothness factor was used to avoid any loss of information. The probability density functions of the elevation of both the cloud points and the mapped data were compared to assess the applicability of the fitted surface. Figure 3 shows the histograms of cloud and gridded data for examples of (a) fine and (b) coarse sand specimens. The values are very similar, indicating that the fitted surfaces do not distort the information obtained from the raw data.

### 2.5. Surface Analysis

Three methods were implemented to characterise the fracture surfaces in this work.

#### 2.5.1. Amplitude Parameters

**Figure 4:**
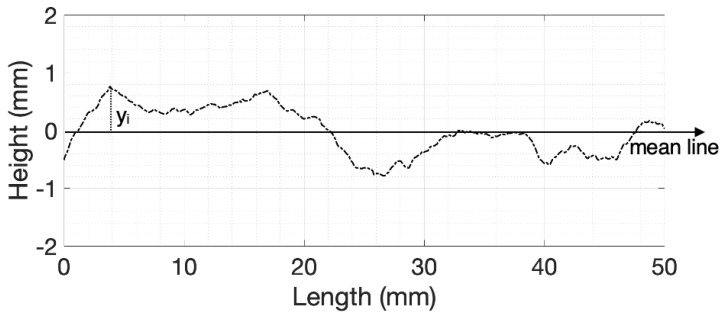
1D example of a normalised height profile and the characteristics used to calculate surface morphological parameters.

The morphology of the surface was characterised through amplitude parameters referring to the distance from the nominal surface [33]:

- arithmetic average height or CLA, $Ra=\frac{1}{n}\sum_{1}^{n}|y_{i}|$;
- root mean square (RMS) roughness, $R_{q}=RMS=\sqrt{\frac{1}{n}\sum_{1}^{n}y_{i}^{2}}$;
- maximum height of peak $R_{p}$ defined as the maximum height above the mean line within a predefined length;
- maximum depth of valleys $R_{v}$ defined as the maximum height below the mean line within a predefined length;
- largest peak to valley height $R_{pv}$ defined as the vertical distance between the highest peak and the lowest valley within a predefined profile length;
- skewness, $Rsk=\sqrt{\frac{1}{nR_{q}^{3}}\sum_{1}^{n}y_{i}^{3}}$;
- kurtosis, $Rsk=\sqrt{\frac{1}{nR_{q}^{4}}\sum_{1}^{n}y_{i}^{4}}$.

where *n* is the number of points and $y_{i}$ is defined as the distance from the nominal surface (mean line) based on the example profile given in Figure 4.

All these quantities examine only the large-scale variations of the surface profile, which may not always be applicable for fracture surfaces in rocks. Myers [37] proposed the RMS of the first derivative of the surface profile ($Z_{2}$) as a way to capture the local smoothness of the profiles. Tse and Cruden [38] investigated the correlation of several surface roughness parameters with JRC and found that $Z_{2}$ had a strong correlation with JRC confirming the hypothesis that this parameter is more suitable for rock surfaces. The first derivative of RMS, $Z_{2}$, is defined as:

(1) $$Z_{2}={\left[\frac{1}{L}\int_{0}^{L}{\left(\frac{dy_{i}}{dx}\right)}^{2}dx\right]}^{\frac{1}{2}}$$

where *L* is the length of the profile and $\frac{dy_{i}}{dx}$ is the first derivate of the height with respect to the length at a specified point (see Figure 4 for graphical representation of the quantities).

The 2D discretised version of this coefficient is defined as:

(2) $$\begin{array}{c} Z_{2}=[\frac{1}{L_{x}L_{y}}[\sum_{i=1}^{}\sum_{j=1}^{}\frac{{\left(z_{i+1,j+1}-z_{i,j+1}\right)}^{2}+{{\left(z_{i+1,j}-z_{i,j}\right)}^{2})}^{2}}{x_{i+1,j+1}-x_{i,j+1}+x_{i+1,j}-x_{i,j}}\\ +\sum_{i=1}^{}\sum_{j=1}^{}\frac{{\left(z_{i+1,j+1}-z_{i,j+1}\right)}^{2}+{{\left(z_{i+1,j}-z_{i,j}\right)}^{2})}^{2}}{y_{i+1,j+1}-y_{i,j+1}+y_{i+1,j}-y_{i,j}}{]]}^{\frac{1}{2}} \end{array}$$

where *x* and *y* define the position of the point under examination, *z* is the elevation or height of the point, $L_{x}$ and $L_{y}$ are the lengths of the surface in the *x* and *y* direction respectively [39].

#### 2.5.2. Analysis in the Frequency Domain

Roughness characteristics can also be examined in the frequency domain. The power spectral density (PSD) reveals information about the texture of the surfaces. The Wiener–Khinchin theorem states the spectral power density is the Fourier transform of the autocorrelation function [61,62]:

(3) $$PSD_{1D}=\int_{-\infty }^{+\infty }\gamma \left(\tau \right)e^{-i\omega \tau }d\tau$$

where the autocorrelation function is defined as the convolution integral expressing the amount of overlap of the function with itself shifted by τ,

(4) $$\gamma \left(\tau \right)=\langle y\left(t\right)y{\left(t-\tau \right)}^{*}\rangle =E\left[y\left(t\right)y{\left(t+\tau \right)}^{*}\right]$$

and y(t) is the function under consideration. In this case, the function is the interpolated fracture surface from the Brazilian tests, where the power spectral density (PSD) provides a decomposition of the surface into its frequency components. Since the function under consideration is discrete, the generalised definition of the discrete PSD was used:

(5) $$PSD_{1D,discrete}=\frac{\Delta s}{L}|\sum_{i=1}^{N}y_{i}e^{-\iota \omega \Delta s}{|}^{2}$$

where *s* represents space, rather than time, *L* is the length of the ‘scan’, *N* is the total population of the points and $y_{n}$ is the height at a given point n.

Similarly, the 2D power spectral density (PSD-2D) is defined as the Fourier transform of the 2D autocorrelation function.

#### 2.5.3. Wavelet Analysis and Decomposition

Another method that can be used effectively to characterise the surface roughness of the samples is wavelet analysis. As in Fourier analysis, it decomposes a signal into a summation of basis functions. A wavelet is a wave-like oscillation defined as a limited duration waveform with zero average value but nonzero norm [63]. Groups of wavelets are generated by shifting and re-scaling the ‘base’ wavelet. The main difference between wavelet and Fourier analysis is that the former deals with wavelets (form of translations and dilations of a finite function called the mother wavelet) while the latter one uses trigonometric polynomials [49].

The wavelet transform holds both spectral (frequency) information and information about the event in time (spatially coordinated in 2D), while the Fourier transform contains only the frequency information. A wavelet technique termed harmonic wavelet transform was developed originally for time–frequency mapping. The method has applications in vibration engineering, geotechnical centrifuge testing, bending wave transmission in a beam, etc. [64]. This wavelet-based linear transform converts a function into a time–frequency representation. The harmonic wavelet transform was used in this investigation replacing time with space to map the roughness of the surface in the frequency domain.

**Figure 5:**
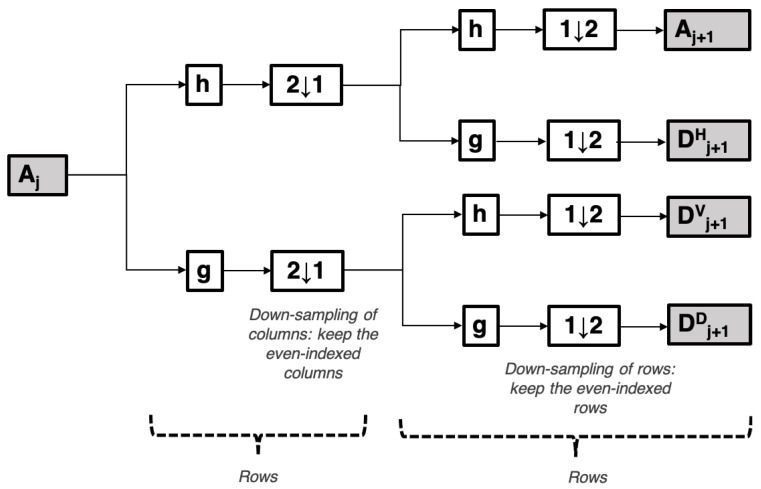
Two-dimensional Discrete Wavelet Transform: schematic of decomposition steps.

Wavelet analysis also allows to account for roughness at different scales. Many types of wavelets exist and a signal is decomposed into multiple bandwidths through filtering and down-sampling. Any discrete function can be represented as a weighted summation of wavelets, with the aid of a Discrete Wavelet Transform (DWT) algorithm. For example, the 2D approximation $A_{j}$ at level *j* shown in Figure 5, is subjected to a two-channel filter phase (a low pass filter, h, and a high pass filter, g) together with down-sampling taking place first in rows and then in columns resulting in the approximation $A_{j+1}$ and the details $D_{j+1}^{H}$, $D_{j+1}^{V}$ and $D_{j+1}^{D}$ [65].

The process is recursive and a 2D signal *S*(*x*, *y*) is decomposed at κ levels into an approximation containing coarse elements (*A*) and details (*D*), which gives:

(6) $$S\left(x,y\right)=A_{\kappa }+D_{\kappa }^{H}+D_{\kappa }^{V}+D_{\kappa }^{D}+D_{\kappa -1}^{H}+D_{\kappa -1}^{V}+D_{\kappa -1}^{D}+\ldots +D_{1}^{H}+D_{1}^{V}+D_{1}^{D}$$

The details, *D*, have physical directions: horizontal, vertical and diagonal, while *x* denotes the level of decomposition. $D_{x}$ corresponds to the largest scale, and in the frequency domain, the lowest-frequency bandwidth information, and $D_{1}$ represents the smallest scale in space or the highest frequency bandwidth. $A_{x}$ is the low frequency profile distortion. The process is reversible; the surface can, therefore, be synthesised based on the approximation and detail functions.

The 2D wavelet decomposition can be explained with the aid of Figure 5, where the approximation $A_{j}$ at level *j* is subjected to two channel filter phases (a low pass filter, h, and a high pass filter, g) applied first to rows and then to columns, resulting in the approximation $A_{j+1}$ and the details $D_{j+1}^{H}$, $D_{j+1}^{V}$ and $D_{j+1}^{D}$.

## 3. Results and Discussion

### 3.1. Tensile Strength

**Figure 6:**
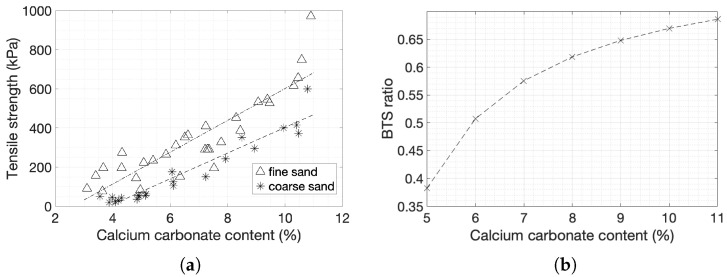
(**a**) Tensile strength with respect to degree of cementation (**b**) BTS of coarse sands over BTS of fine sands with respect to degree of cementation.

The results of the indirect tensile strength tests are plotted against the degree of cementation (Figure 6a). At a cementation level of 3%, the tensile strength of fine sands was around 50 kPa, and this increased to about 700 kPa at 11% cementation, the highest cementation level reached with this MICP protocol. Coarse sands showed a tensile strength of about 25 kPa at 4% cement by weight, which increased to 500 kPa at 11% cementation.

A linear regression provided a best fit for both types of sands. The rate of change in tensile strength of fine sands was slightly higher compared to that of coarse sands, which also had overall lower strength. The ratio of the tensile strength (BTS) of coarse sands to the BTS of fine sands was 40% at low cementation and increased to 70% at the highest cementation levels.

Straight line fits to the data for fine and coarse sands respectively are:

(7) $$BTS=81.274x-211.21\;R^{2}=0.7921$$

(8) $$BTS=65.9668x-252.27\;R^{2}=0.9179$$

where *x* is the cementation level measured as a percentage.

The unconfined compressive strength (UCS) for coarse and fine sands, prepared with identical protocols, was examined by Konstantinou et al. [13]. Both sands showed a substantial increase in strength when the cementation increased and an exponential curve best described the rate of change of strength with the degree of cementation. The regressions for fine and coarse sands are:

(9) $$UCS=56.911e^{0.4018x}$$

(10) $$UCS=56.544e^{0.3568x}$$

**Figure 7:**
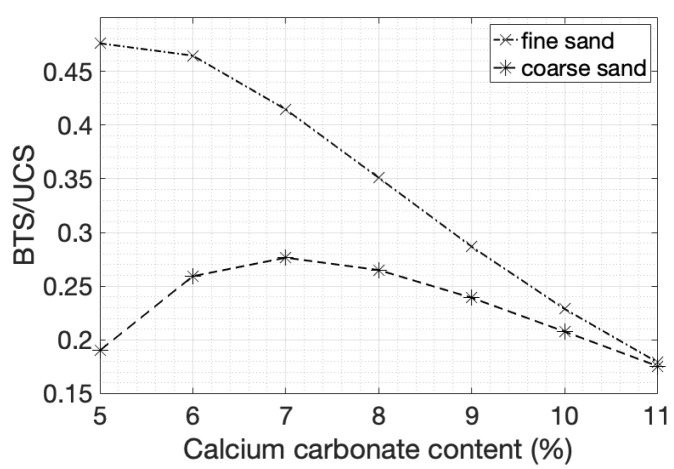
BTS-to-UCS ratio of fine and coarse sands with respect to the degree of cementation.

The ratio of tensile to compressive strength of fine sands (Figure 7) dropped from 50% to 25% as the cementation level increased, while for coarse sands the ratio remained in the range of 20% to 25%. The tensile strength of natural soft sandstones is estimated as 10–40% of UCS, with most of the values falling in the range between 10 and 20% [66].

### 3.2. Fracture Surfaces and Their Roughness Characteristics

**Figure 8:**
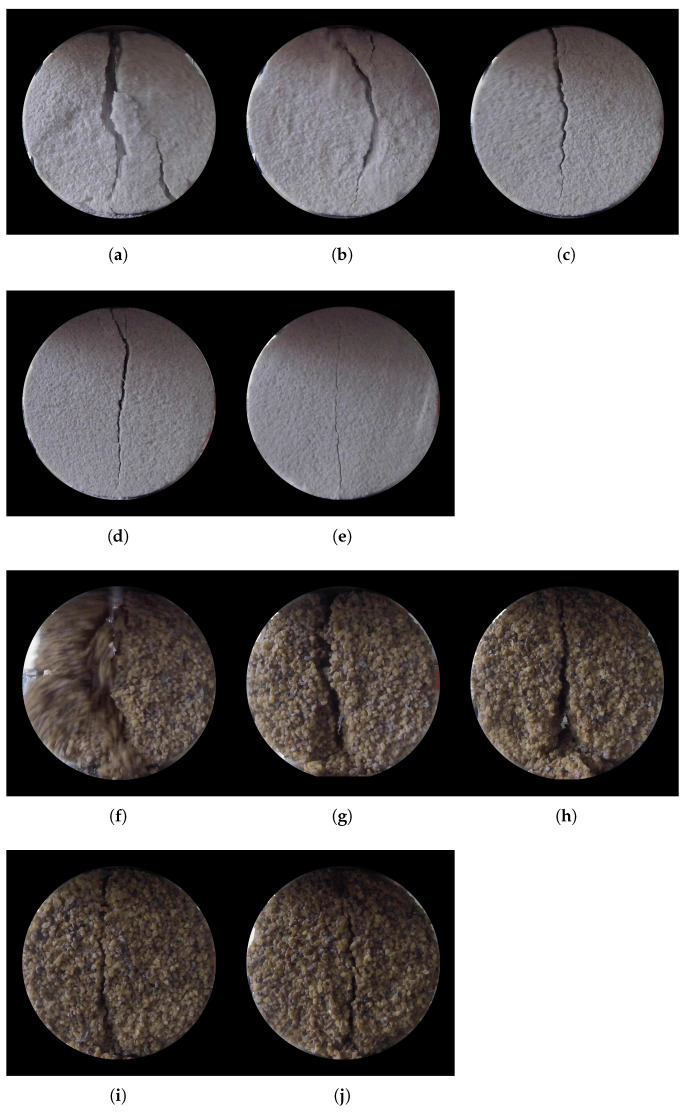
Final view of fracture on the front of the specimen; (**a**–**e**) from fine sands from weakest to strongest specimen (cementation levels at 3.1%, 3.4%, 5.9%, 9.1% and 10.9%); (**f**–**j**) from coarse sands from weakest to strongest specimen (cementation levels at 3.9%, 6.1%, 7.9%, 8.5% and 10.8%).

Figure 8 shows the fractures in ten specimens of fine and coarse sands at the end of the test. The measured tensile strength and cementation level increased from left to right. As the degree of cementation increased, the failure became mainly tensile with shear only in the vicinity of the contacts with the top and bottom pedestals of the loading frame where the fracture took the shape of a curved line. The fracture was confined to the mid-plane of the specimens with the stronger of fine sands showing straight vertical failure planes indicating pure tensile mode fracture. Weaker fine sands, instead, showed multiple fractures with deviations from the central vertical line. The effect of inhomogeneities was more pronounced in the weaker specimens, as the fracture propagated along a path connecting weaker clusters of cement. This phenomenon faded out as the cementation level increased and fewer weak areas were left in the specimens. It was shown by Konstantinou et al. [13] that the uniformity of the samples increases as the carbonate concentration increases. The authors reported that variance in the measurements of carbonate amount declined as the cementation level increased, with largely uniform samples at high levels of cementation, around 10%. The carbonate precipitation was relatively uniform for fine sand even at lower cementation levels. No particular trend was observed in the cementation profiles across the height of each sample.

In the tensile test, weak coarse sands tended to disaggregate at the grain scale and showed evidence of shear failure adjacent to the loading points. Detachment of particles was evident and cracks followed a path from weak cluster to weak cluster of cement. Individual sand grains are stronger than either the cement or the interface bond with the particles, and thus fractures followed bond–grain boundaries or occurred directly across cement crystals.

**Figure 9:**
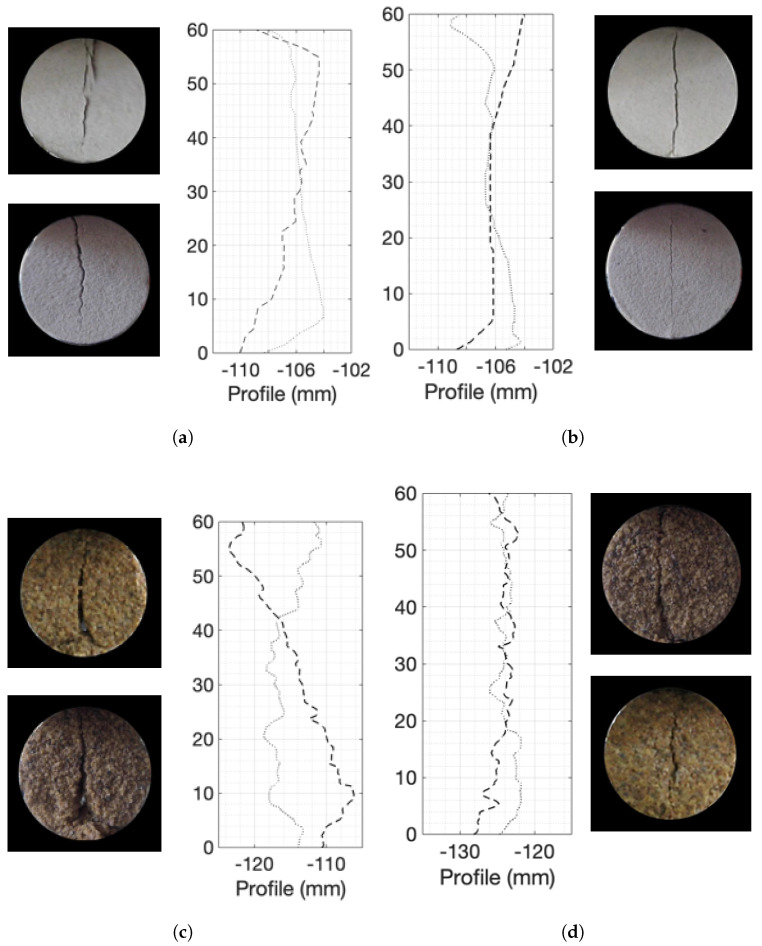
Fracture profiles on the two edges of four specimens: (**a**) a weak fine sand specimen of 5.85% cementation; (**b**) a strong fine sand specimen of 9.1% cementation; (**c**) a weak coarse sand specimen of 7.9% cementation; (**d**) a strong coarse sand specimen of 10.8% cementation.

The variability observed on the face of the fractures also extended to the direction perpendicular to the view of the camera (*y*-direction as defined in Figure 2). Figure 9 shows the profiles of the fractures on both sides of four specimens: a weak and a strong fine sand and a weak and a strong coarse sand. The fractures propagated to the faces of the specimens at different times and are clearly not the same.

Many visual observations can be made based on these figures. The largest differences observed in the profiles between the two visible faces occurred in the weak coarse samples. Both the weak and the strong fine sand samples showed more consistent profiles. The coarse-grained specimens seemed to have higher roughness compared to the fine-grained ones. This was expected because the failure surface had to go around the grains and larger grains would create a more craggy surface. Based on the observations of the end of the specimens, the fracture surfaces are also likely to show variations along the length of the specimens, especially for weaker materials.

These observations indicate that the cementation level, therefore the strength of the material, determines the fracture growth and its final shape in the indirect tensile strength tests. The fracture in weaker materials tended to deviate more from the vertical than in the stronger materials. The spatial distribution of the cement within the granular matrix and the particle grain sizes also affected the fracture growth, but had a secondary effect.

Although the photos of the specimens and the end profiles of the fractures provided some indications of the general behaviour, additional insight could be acquired with more systematic characterisation of the 3D surfaces. Linear scans of the failure surfaces were taken in the *x*-direction for a 50 mm length in the centre of the specimens, at equal distance from the ends. Additionally, the surface profiles were segmented into patches of dimension 50 × 10 mm, with the larger dimension oriented in the *x*-direction and the shorter dimension in the *y*-direction, acquired along the *z*-axis of the specimens (see Figure 2 for coordinate system). The weaker specimens, both fine and coarse, disaggregated along the contacts with the pedestals. The resulting fracture surfaces could only be acquired in the central portion. In order to be consistent in the comparisons, scans of 50 mm length were examined for all specimens, irrespective of the state of the outer 10 mm on either side.

**Figure 10:**
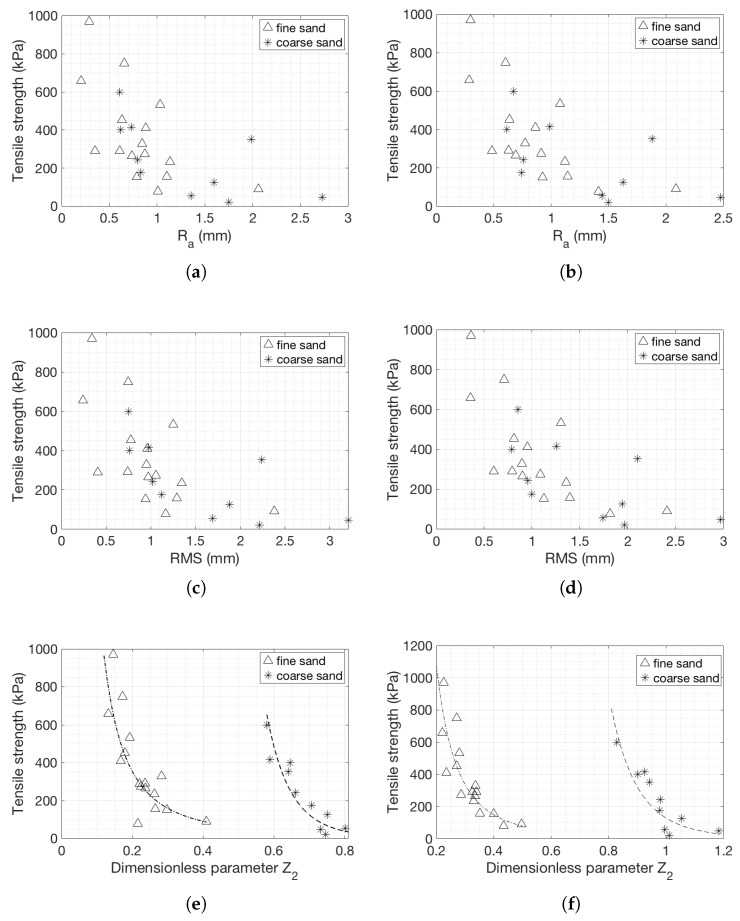
Tensile strength with respect to: (**a**,**b**) $R_{a}$—Arithmetic average height parameter for a 50 mm scan at the centre of the specimen and for a 2D 50 × 10 mm window, respectively; (**c**,**d**) RMS—Root mean square roughness for a 50 mm scan atthe center of the specimen and for a 2D 50 × 10 mm window, respectively; (**e**,**f**) $Z_{2}$—Dimensionless roughness parameter for ta 50mm scan the center of the specimen and for a 2D 50 × 10 mm window.

The more appropriate metrics to describe the surface characteristics were the arithmetic average height, the root mean square roughness and the dimensionless parameter $Z_{2}$, as shown in Figure 10 for both the line scans at the centre and for the 2D windows.

The arithmetic average height is commonly used to assess height variations. As discussed previously, it describes the variations of the surface at a larger scale. For both coarse and fine sands, a decrease in the tensile strength results in an increase in the value of RMS (Figure 10a). The smallest variation of the height is observed for the strongest sample, the fine sand with a tensile strength of 970 kPa, while the highest height variation is for coarse sands with the lowest strength of 20 kPa. For fine sands, and for tensile strength in the range of 250 and 450 kPa, the data are scattered.

The arithmetic average data for the window measurements are similar to the line scans, although with larger variations (Figure 10b). The measurements from a single line are representative of the full window, at least when taking into consideration the arithmetic mean deviation of the profiles.

The results obtained for both types of sands show that the RMS decreases when the strength of the specimen is higher for both window and line scans demonstrating consistency (Figure 10c,d). The RMS parameter does not seem to be able to differentiate the tensile strength of very strong fine sand specimens as it provides very similar values.

The amplitude parameters show some dependency on the tensile strength. RMS and average roughness provide rough trends. The two parameters describe large-scale variations neglecting any local effects that might exist and they are not affected by the sudden height changes at the edges of the samples. The tensile strength is plotted against the dimensionless parameter $Z_{2}$ in Figure 10e,f. The dimensionless characteristic $Z_{2}$ describes the local smoothness or roughness of a profile, eliminating low frequencies, and thus ignoring large-scale variations.

The fit is a power curve for both sands and both linear and 2D patch measurements. An increase in strength resulted in an increase of this parameter. As expected, the dimensionless parameters for the coarse sand specimens are greater than for the fine sand specimens, and they exhibited larger changes when the tensile strength changed. The generated fracture needs to go around the grains, therefore it causes larger $Z_{2}$ values. The ranges of the $Z_{2}$ values are very different for the window and line scans in the case of coarse sands while for fine sands the values are very similar (between 0.15 and 0.4).

In both sands and in both 1D and 2D representations, the parameter $Z_{2}$ is considered to be the most appropriate on the basis of the R-squared values of the fits compared to the CLA and RMS in both line scans and window measurements, although all parameters tend to show good correlation with the resulting strength. The fittings for fine and coarse sands for the 50 mm line scans (Equations (11) and (12)) and 50 × 10 mm windows for fine and coarse sands (Equations (13) and (14)) are listed below:

(11) $$BTS=14.997Z_{2}^{-1.964}\;R^{2}=0.6199$$

(12) $$BTS=5.3593Z_{2}^{-8.819}\;R^{2}=0.7269$$

(13) $$BTS=10.708Z_{2}^{-2.864}\;R^{2}=0.839$$

(14) $$BTS=128.54Z_{2}^{-8.725}\;R^{2}=0.5538$$

**Figure 11:**
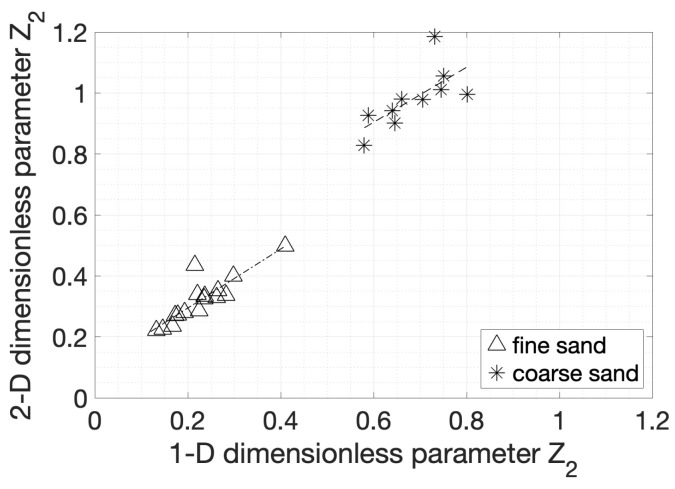
The 2D dimensionless parameter $Z_{2}$ is plotted against the 1D dimensionless parameter $Z_{2}$.

Since the $Z_{2}$ parameters provided better fits compared to the rest of the amplitude parameters, they are further analysed. The 1D and 2D $Z_{2}$ parameters are plotted against each other in Figure 11 to assess whether the 1D parameters are sufficiently representative of the surfaces. Both sets of data show strong correlation, indicating that $Z_{2}$ for the 1D case may be representative of the whole surface, even if the actual values differ because of larger variations included when taking more points into account. As the grain size increased, the trend deviated from the 1:1 line, which means that the 1D scan became less representative of the whole surface.

Roughness parameters are often determined by analysing the results in the frequency domain. A powerful tool is the power spectral density (PSD). One of the greatest advantages of PSD is the fact that it contains information at various scales. As discussed earlier, the generated fracture surfaces are characterised by a macro-scale fracture shape (form of the surface) and by local roughness, which is dependent to a great extent on the grain size. The analysis in the frequency domain provides a way to separate the different scales and enables a more precise interpretation of the results. Only the metrics that provided meaningful results are shown in this section.

**Figure 12:**
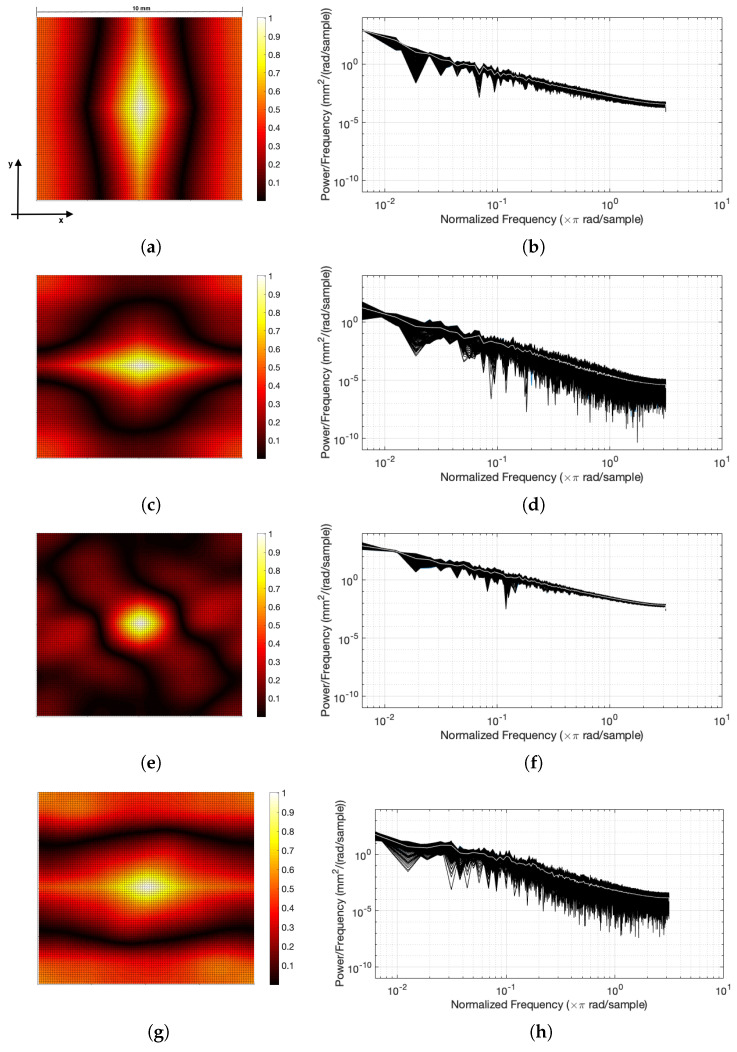
Low and high tensile strength 2D autocorrelation functions and 1D PSD profiles for fine sands (**a**–**d**) at cementation levels of 3.1% and 10.9%, respectively, and of coarse sands (**e**–**h**) at cementation levels of 3.9% and 10.8% respectively.

Patches with a 10 × 10 mm size were extracted from the centre of each failure surface and the 2D autocorrelation functions were constructed. Autocorrelation for profiles from specimens with low, medium and high tensile strength are shown in Figure 12 for fine and coarse sands. (Figure 12). As shown in the contour plots, the weak samples’ autocorrelation function decayed more quickly along the *x*-axis, because of the larger profile heights variation.

The power spectral density was calculated for multiple 1D scans (length of 50 mm) in the direction of the applied load to assess the roughness characteristics at both low (shape of fracture) and high frequencies (local roughness). The width of the window was 10 mm in the *y*-direction, therefore, a total of 200 scans were evaluated. Power spectral densities for profiles from specimens with low, medium and high tensile strength are shown in Figure 12 for fine and coarse sands. The dark area shows all the scans taken from the surface and the white line is the average of the power spectral densities. Generally, the rougher a surface is, the higher the power spectral density is [67]. As the sample’s strength increased, the power spectral density was lower across the whole range of frequencies, indicating smoother samples or samples with a less rough fracture. This holds for both fine and coarse sands. However, the difference between strong and weak specimens is more evident when looking at the higher frequencies where the variations are more significant (larger scales compared to the grain size), suggesting that the strength of the sample dictates the way the specimen breaks during the Brazilian test. The analysis in the frequency domain provided useful information only when the results were analysed in the direction of the applied load (defined as the *x*-direction in the coordinate system in Figure 2).

**Figure 13:**
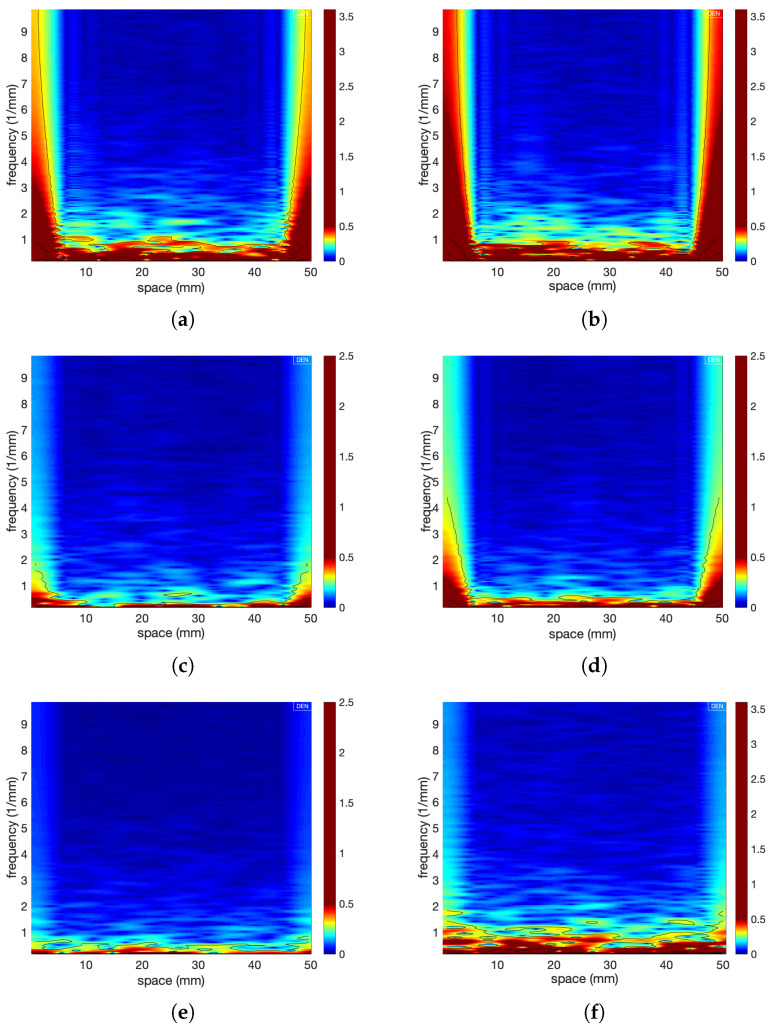
Low, medium and high tensile strength wavelet profiles for fine sands (**a**,**c**,**e**) at cementation levels of 3.1%, 7.3% and 10.9%, respectively, and for coarse sands (**b**,**d**,**f**) at cementation levels of 3.9%, 8.5% and 10.8%, respectively.

Wavelet transform provides information on both the frequency content of a profile and the variation of the frequency content in space. The profiles were analysed with the aid of the harmonic wavelet transform. Figure 13 represents a contour map of the three-dimensional surface obtained by plotting the magnitude of the wavelet coefficients against location and frequency. The measurements were obtained for 1D line scans of the failure surfaces taken along the diameter of each specimen for a length of 50 mm (*x*-direction as defined in Figure 2). Six scans are shown, three for fine sands ((a) low, (c) medium and (e) high strength) and three for coarse sands ((b) low, (d) medium and (f) high strength). In these wavelet spectra, the blue colour represents the lowest wavelet component and the red colour represents the highest wavelet component.

For both weak fine and coarse sands, the magnitude of the wavelet coefficients was greater over a larger range of frequencies (0–4 and 0–91 mm${}^{-1}$, for fine and coarse sands, respectively) at the edges of the scans (0–5 mm and 45–50 mm), whilst stronger samples had wavelet coefficients of approximately the same magnitude across the whole length. The frequencies observed at these locations show clearly that the elevations of the points constituting the profile under examination (for example, see Figure 4) varied greatly. The higher variations in profile height at the edges of fracture face images indicate the location of shear failure. The stress distribution in stronger samples was more uniform as shown by the figure: the magnitude of the wavelet component was similar through the profile of the sample.

### 3.3. Wavelet Decomposition for Elimination of Particle Size Effects

**Figure 14:**
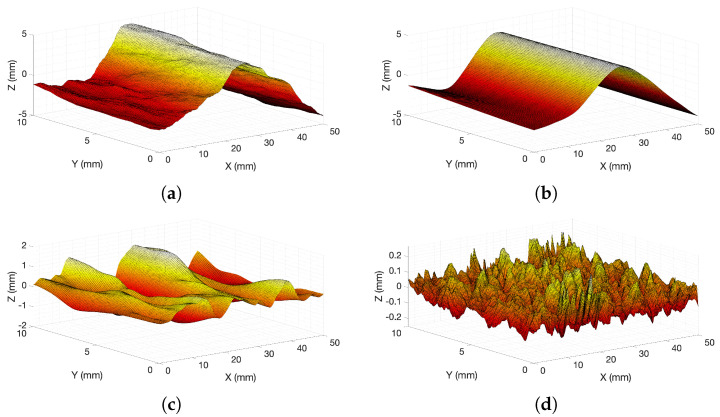
Wavelet decomposition of an example generated surface (2D 50 × 10 mm window) by testing a fine sand specimen at a cementation level of 3.1%: (**a**) example surface, where the generated failure surface from Brazilian test is on the *x*–*y* plane; (**b**) form of the surface; (**c**) waviness of the surface; (**d**) roughness of the surface.

This analysis aimed to isolate high-frequency changes and low-frequency changes (i.e., slope vs. a dithering) to bridge the differences across the scales (fine and coarse sands) and then perform surface analysis on each of those ranges. The digitised surfaces (2D 50 × 10 mm windows) were decomposed into different scales in order to calculate the roughness at different levels. Three levels of decomposition were considered (a decomposition example is shown in Figure 14). The first one captured the form of the surface at the larger scale (corresponding to a variation of approximately 7 mm for fine sands and 10 mm for coarse sands), corresponding to the overall shape of the fracture (Figure 14b). The second level of decomposition described the waviness (corresponding to a variation of approximately 3 mm for fine sands and 4 mm for coarse sands) (Figure 14c), and the third one—the roughness (corresponding to approximately a variation of 0.5 mm for fine sands and 2 mm for coarse sands), (Figure 14d).

The decomposition was carried out with the aid of the reverse bi-orthogonal filter (ReverseBior6.8 mother wavelet family function) due to its good linear transmission properties [65]. The waviness was represented by coefficients corresponding to (Detail 8 + Detail 7 + Detail 6) of the decomposition, while roughness was reconstructed with Detail 1 to Detail 5. Further information on the decomposition can be found in the study by Zou et al. [65].

**Figure 15:**
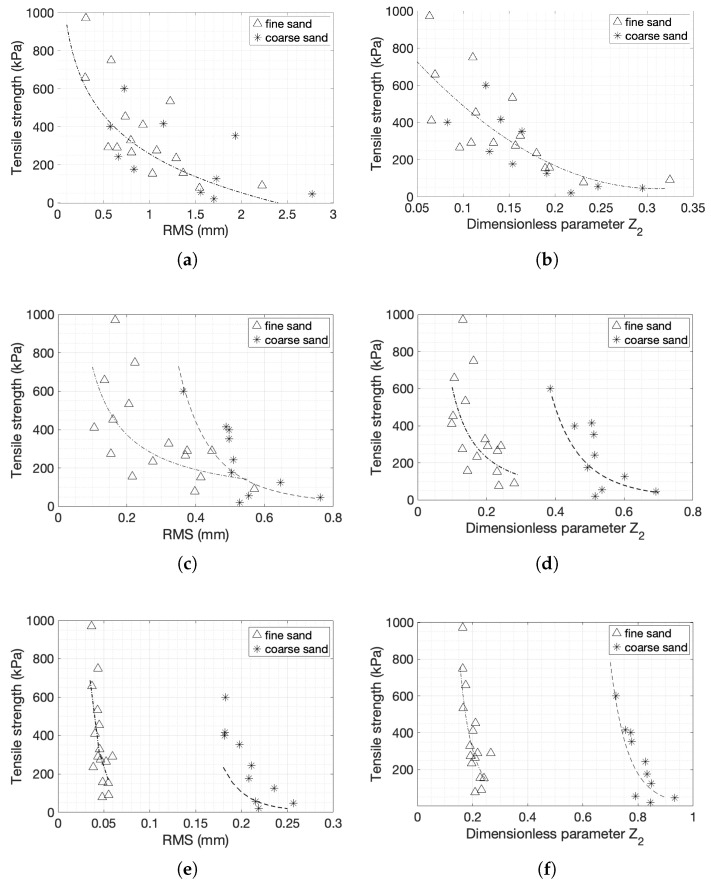
(**a**,**b**): RMS and $Z_{2}$, respectively, for the reconstruction representing the form of the surface; (**c**,**d**) RMS and $Z_{2}$, respectively, for the reconstruction representing the waviness; (**e**,**f**): RMS and $Z_{2}$, respectively, for the reconstruction representing the roughness.

RMS and $Z_{2}$ were calculated for each component of the deconstructed surface (Figure 15). Interestingly, almost all cases showed correlations with all three levels of decomposition, including the form of the surface. The shape of the fracture surface correlates well with the tensile strength of the specimen. As discussed previously, the stronger the specimen was, the straighter the failure line was, giving purely tensile fracture (when the samples were weaker, the failure path was governed by the locations of the weaker clusters). Therefore, the form of the surface had a good correlation with the roughness parameters. The grain size effects were removed at the larger scale, making the results between fine and coarse sands directly comparable. The larger-scale shape of the failure surface seems to relate solely to the degree of tensile vs. shear failure, which correlates with strength and cementation.

The parameters calculated from the waviness-level surfaces (see Figure 15b) show that the trends are clearly dependent on the grain sizes. The strength of the specimen also plays an important role. The frequency ranges that correspond to roughness (see Figure 15c) have no correlation to the roughness parameters, since these are closely related to the concept of white noise in this application.

### 3.4. Correlations with the Carbonate Content

**Figure 16:**
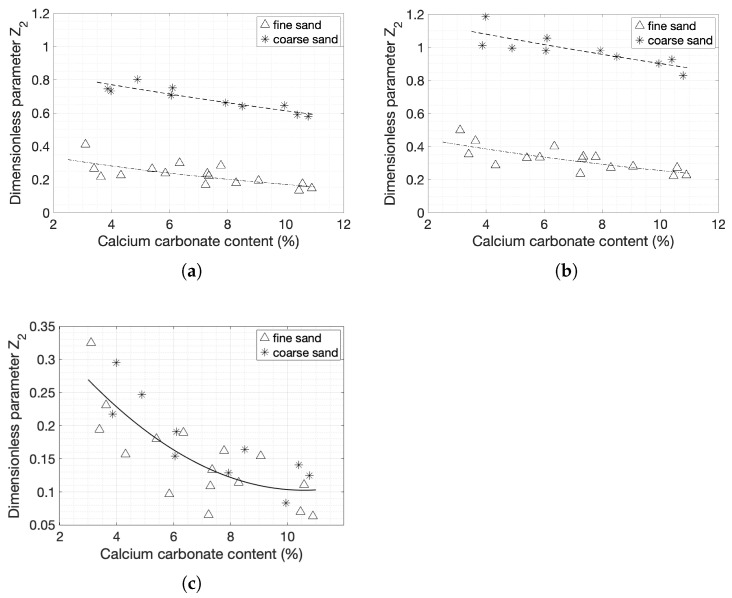
(**a**) Dimensionless parameter $Z_{2}$ for the 1D linear scans with respect to the carbonate content; (**b**) Dimensionless parameter $Z_{2}$ for the 2D 50 × 10 mm windows with respect to the carbonate content; (**c**) Dimensionless parameter $Z_{2}$ for the 2D 50 × 10 mm decomposed windows at the form of the surface levels, with respect to the carbonate content.

The amplitude parameters that provided the best fits were also correlated to carbonate content. Exponential correlations were observed between the 1D and 2D $Z_{2}$ parameter and the degree of cementation (Figure 16a,b). The surface roughness of coarse sand specimens decreased more rapidly as the degree of cementation increased compared to fine sand specimens, explaining the higher R-squared value obtained for coarse sands. The R-squared values of coarse sands were 0.8309 and 0.6779 for 1D and 2D $Z_{2}$, respectively, while the R-squared values of fine sands were 0.5048 and 0.5807 for 1D and 2D $Z_{2}$, respectively. The specimen split into two identical parts with locally flat surfaces. Fine sands exhibited less decrease in $Z_{2}$ with increasing cementation level. Since this parameter takes into account local variations in height, the correlation shows that clusters of grains and cement are less common in specimens with higher cementation levels samples and fewer inhomogeneities were present [13]. Figure 16c depicts the relation between the 2D dimensionless coefficient $Z_{2}$ for the form of the surface as calculated previously and the degree of cementation. The results were more scattered in this case and the fitting was a curve rather than a line, with an R-squared value of 0.5604. However, both types of sands were comparable and a single regression equation could be derived to correlate $Z_{2}$ and degree of cementation without any effects of the grain size.

## 4. Conclusions

The MICP products can be successfully used as a substitute for natural weakly cemented carbonate sandstones in laboratory studies with tests involving complex loading conditions. The novelties of this research are: (i) the measurement of tensile strength of bio-cementacross two materials differing greatly in grain size across various cementation levels, (ii) the detailed examination and quantification of the generated fracture surface characteristics and (iii) the correlation of the derived surface metrics with the tensile strength of the specimens.

An increase in strength was obtained when the cementation level increased for both sands; however, the change was more evident in fine sands. The ratio of tensile strength over the unconfined compressive strength resembled the values obtained in competent rocks only at the highest cementation levels, indicating that the addition of cementation transitioned the material from soil to weak rock.

The relation between unconfined compressive strength and tensile strength is particularly important, as it is often needed to identify tensile or shear failure. These effects were further studied with the assessment of the textural characteristics of the generated fracture faces. At lower cementations, the surfaces presented higher variations in height because of the presence of shear failures. The height variations became gradually lower with increasing cementation levels, as the specimens became stronger. The fractures in those cases were less rough.

Several amplitude parameters describing roughness of the failure surface were examined. The dimensionless parameter $Z_{2}$ provided best fits for both 1D and 2D scans as it can better account for localised effects. The analysis in the frequency domain led to similar conclusions, although it was also possible to separate features across various scales. Wavelet analysis allowed to map the wavelet magnitudes with space and frequency, providing further indication that shear failure was present mainly at the edges of the disk specimens.

The grain size effects did not allow for direct comparison of the results across the fine- and coarse-grained sands. Bridging of the scales was achieved via wavelet decomposition and reconstruction of surfaces. The primary shape of the surface was defined by removing the grain sizes, which was proven to be directly related to the mode of failure, and hence the tensile strength.

For future work, machine learning (ML) could be used on the current experimental dataset to develop models that mimic the tensile strength and the correlations obtained experimentally. The ML algorithm would give the chance of generating synthetic and realistic data points that could allow further investigation of the correlations and relationships obtained in this study.

## Figures and Tables

**Table 1:** Summary of the tests.

	Average Particle Size (mm)	Cementation Level Range (%)	No of Tensile Strength Tests	No of Fracture Surfaces Being Analysed
Fine sands	0.18	3.1–10.9	30	16
Coarse sands	1.82	3.55–10.78	22	10

## Data Availability

The data presented in this study are available upon reasonable request from the corresponding author.
